# Fatal Intracranial Hemorrhage Associated with Oral Warfarin Use

**DOI:** 10.7759/cureus.3571

**Published:** 2018-11-12

**Authors:** Amado Jimenez-Ruiz, Alejandro Gutierrez-Castillo, José Luis Ruiz-Sandoval

**Affiliations:** 1 Neurology, Instituto Nacional de Ciencias Medicas y Nutricion Salvador Zubiran, Ciudad de Mexico, MEX; 2 Miscellaneous, Instituto Tecnológico Y De Estudios Superiores De Monterrey, Monterrey, MEX; 3 Neurology, Hospital Civil De Guadalajara "Fray Antonio Alcalde", Guadalajara, MEX

**Keywords:** stroke, cerebrovascular disease, intracranial hemorrhage, warfarin, anticoagulation, coagulopathy, atrial fibrillation, fluid-fluid level, blood-fluid level, neurocritical care

## Abstract

Intracerebral hemorrhage is the most devastating complication in patients taking oral warfarin. Despite theoretic reversibility with fresh frozen plasma, vitamin K and prothrombin complex concentrate, it remains an entity with high mortality. Fluid-fluid level, also known as blood-fluid level, sedimentation level, or hematocrit effect, seen on noncontrast computed tomography (CT) scan is a characteristic finding associated with patients who have coagulopathy or who are receiving oral anticoagulation therapy.

We present the case of an 80-year-old female patient requiring long-term anticoagulation due to atrial fibrillation, who presented acute neurological symptoms including thunderclap headache and hemiparesis. Urgent noncontrast CT scan showed classic signs of warfarin-induced intracranial hemorrhage with a fatal outcome a few hours after admission.

## Introduction

Atrial fibrillation (AF), the most common type of sustained cardiac arrythmia, involves the disorganized electrical activity in the atria that leads to a rapid, irregular ventricular contraction [1]. Long-term anticoagulation with oral vitamin K antagonists (VKAs) has been considered as a mainstay treatment for primary and secondary prevention of stroke in this group of patients [2]. Warfarin, the most widely used anticoagulant in the world, has the disadvantages of a narrow therapeutic index and wide interactions with food and other commonly used drugs. This requires a strict monitorization of the international normalized ratio (INR) to maintain its efficacy and safety [3-4]. Warfarin has many adverse effects; however, intracranial bleeding is the most lethal complication [4].

## Case presentation

An 80-year-old female was admitted to the hospital following thunderclap headache. Medical history was relevant for hypertension, hypothyroidism, congestive heart failure, chronic kidney disease, and valvular AF with a prior ischemic stroke. Her current medications included oral warfarin use. Her last known INR was in therapeutic range. The patient was also taking metoprolol, nifedipine, amiodarone, atorvastatin, levothyroxine, and omeprazole.

**Figure 1:**
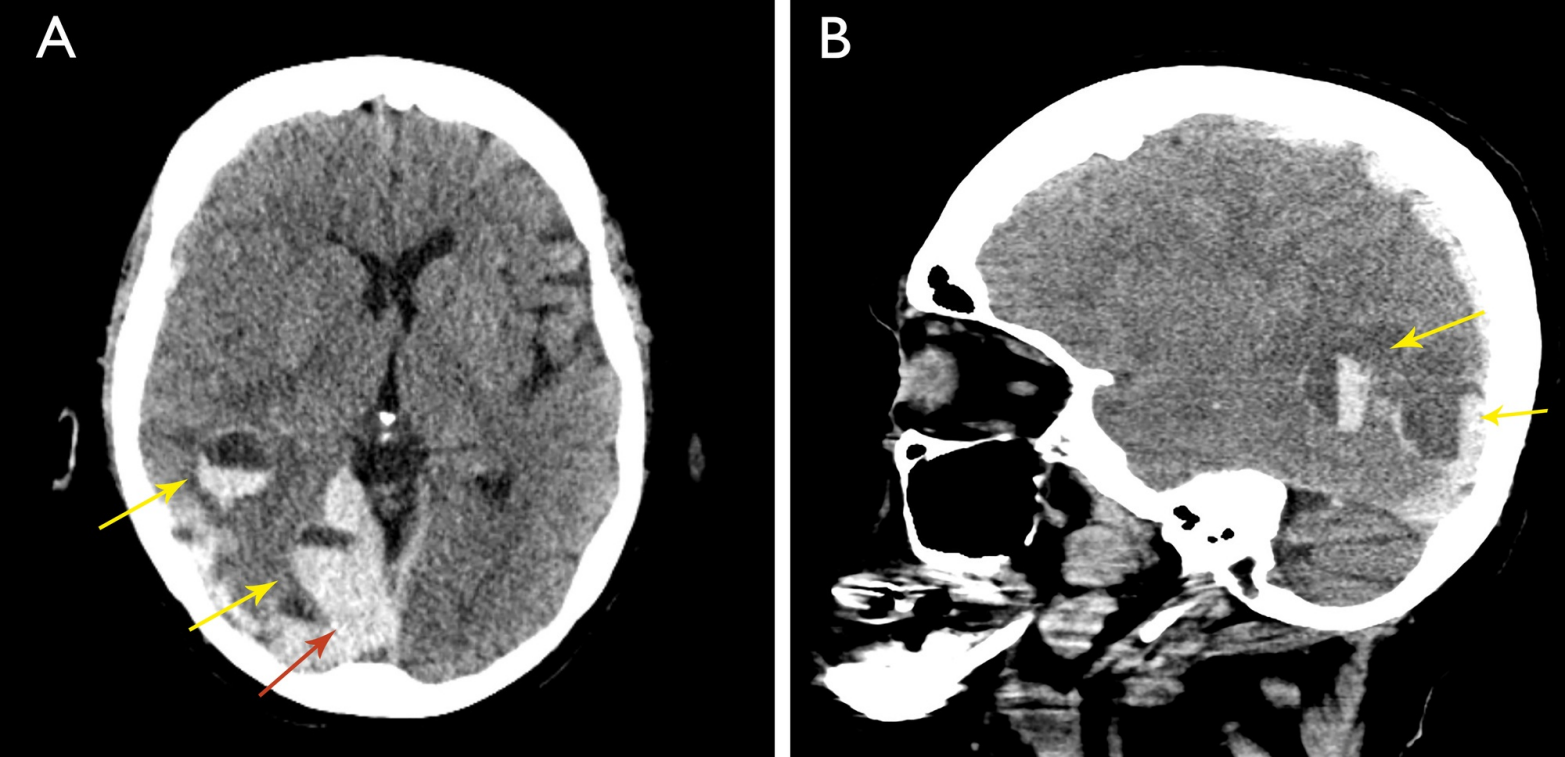
Nonenhanced computed tomography of the brain, with axial view (Panel A) and sagittal view (Panel B), showing an acute supratentorial subdural hematoma (red arrow) and blood fluid levels in right parietal and occipital lobe (yellow arrows).

At admission, general exam showed a slightly increased blood pressure (130/80 mmHg) and irregular tachycardia. Electrocardiogram confirmed AF. Neurological exam was relevant for stupor and left hemiparesis. Urgent noncontrast enhanced computerized tomography (CT) scan revealed the combination of acute supratentorial subdural hematoma and parenchymal hematomas with the formation of several blood fluid levels in right parietal and occipital lobe, with midline shift and compression of the ipsilateral ventricle (Figure 1). Clinical and radiographical findings were highly suspicious for warfarin-induced intracranial hemorrhage.

Laboratory test results were relevant for low hemoglobin of 9.2 g/dL (normal: 14-17 g/dL), prolonged prothrombin time (PT) of 44.20 seconds (normal: 9-12 seconds), prolonged INR of 4.5 (recommended range: 2.0-3.0), and glomerular filtration rate of 9 mL/min. Despite the administration of fresh frozen plasma, vitamin K and prothrombin complex concentrate, the patient became comatose and died eight hours after admission in the intensive care unit. 

## Discussion

Direct oral anticoagulants (DOACs, dabigatran, apixaban, rivaroxaban, and edoxaban) have emerged as important alternatives to VKAs in the setting of stroke prevention, especially in the setting of nonvalvular AF. However, VKAs (warfarin, coumadin) remain the only approved drugs for certain conditions including valvular AF and prosthetic valvular replacement. Warfarin has a narrow therapeutic index that makes it a difficult drug to titrate appropriately. Patients with AF taking warfarin showed that nearly 50% of the time, INR was outside the therapeutic range [5]. An INR over three is associated with an increased risk of bleeding [3], and intracranial hemorrhage remains the most devastating complication. It is also associated with 90% of warfarin-related deaths [6]. Patients who survive this neurological emergency exhibit important long-term disability [6]. In our patient, the last known INR was within normal limits, however, during admission it was above four, which put her at high risk for bleeding complications.

Radiological signs include supratentorial subdural hemorrhage combined with parenchymal hematomas and fluid-blood levels (also called hematocrit effect). These signs are highly sensitive (59%) and specific (98%) for warfarin-induced intracranial hemorrhage [7]. This sign is defined radiologically as the presence of an area of low attenuation on noncontract CT above, and high CT attenuation below with a well-defined line of separation inside an area of intraparenchymal hemorrhage. It is a rare and almost pathognomonic finding denoting active coagulopathy secondary to oral anticoagulant use. It should be seen within the first 12 hours. Although bleeding can occur at any location, the most common site in these patients is supratentorial and intraparenchymal [7]. If present on a noncontrast CT scan, a high index of suspicion for active bleeding associated with anticoagulants should be raised, and emergent action should be undertaken.

In this scenario, the degree of prolongation of INR correlates with diverse negative outcomes including size and growth of hematoma, as well as functional outcome and mortality [8]. Urgent management is required and current practice guidelines include using vitamin K and prothrombin complex concentrate (over fresh frozen plasma) to try and revert the ongoing bleeding. The current goal is to lower INR to less than 1.4 within the first four hours after onset, and blood levels should be monitored every four to six hours in the first 24 hours, and daily for at least five to seven days. Use of the recombinant factor VIIa is not currently recommended as monotherapy [9]. Even though recommended management was given to our patient, she unfortunately had a fatal outcome.

## Conclusions

Vitamin K inhibitors such as warfarin are indicated for primary and secondary prevention of stroke in patients with valvular AF. Warfarin-induced intracerebral hemorrhage remains the most lethal complication of its use, with very high mortality. Specific radiological findings aid in the differential diagnosis and prompt management of this entity that should be treated as a true neurological emergency.
